# A Deep Learning Model for Estimating the Quality of Bimetallic Tracks Obtained by Laser Powder-Directed Energy Deposition

**DOI:** 10.3390/ma17225653

**Published:** 2024-11-19

**Authors:** Vincent Wong, Alberta Aversa, Alessandro Roger Rodrigues

**Affiliations:** 1 DISAT—Department of Applied Science and Technology, Politecnico di Torino, Corso Duca Degli Abruzzi 24, 10129 Torino, Italy; vincent.wong@polito.it; 2 Department of Mechanical Engineering, São Carlos School of Engineering (EESC), University of São Paulo (USP), São Carlos 13566-590, Brazil; roger@sc.usp.br

**Keywords:** bimetallic component, laser powder-directed energy deposition, diffusion, deep learning, Inconel 718, AISI 316L

## Abstract

During the fabrication of Inconel 718–AISI 316L bimetallic components via laser powder-directed energy deposition, understanding the relationships between processes, microstructures, and material properties is crucial to obtaining high-quality parts. Physical–chemical properties, cooling rates, and thermal expansion coefficients of each material may affect the microstructure of parts, generating segregations and cracks. This paper analyzes how the process parameters affect the dimensions, chemical composition, and microhardness of bimetallic tracks. We created a dataset that included laser power, powder feed rate, material, skeletal density, dimensional features, chemical composition, and microhardness. Then, a deep learning methodology using a multilayer perceptron was used to estimate the relationship between these factors. The architecture comprised four inputs in the input layer and five hidden layers with 20, 40, 30, 30, and 30 neurons, respectively. This architecture was used to estimate the dimensional features, chemical composition, and microhardness. The model precision was evaluated using the determination coefficient (R^2^) and the mean absolute error (MAE) function. Lastly, we used a random forest classifier to select the bead quality from the optimal process parameters. The results showed a significant decrease in training loss and validation loss between 50 and 100 epochs. This decreasing trend continued until 350 epochs. This paper contributes to understanding the relationships between process–structure properties in the bimetallic tracks of Inconel 718 and AISI 316L.

## 1. Introduction

One of the greatest potentials of laser powder-directed energy deposition (LP-DED) is the ability to create large components with composition variations that confer local functional performance to the part [1]. This aspect is of interest to many industrial fields. For example, materials with high corrosion resistance, wear resistance, and specific mechanical properties are required in maritime, chemical, and industrial sectors. Materials with specific high temperatures, mechanical properties, and thermal properties are needed in the energy field. These requirements might be difficult to find in a single material, and for of these reasons, the fabrication of multi-material (MM) components can be a suitable alternative [2,3]. The fabrication of bimetallic components combining AISI 316L and Inconel 718 (IN 718) has recently gained a large interest in the industrial field owing to the combination of high temperature resistance, mechanical properties, and corrosion resistance of the Ni–Cr alloy, with the high strength-to-weight ratio, easy machinability, and low density of the stainless steel. The possibility of joining Inconel 718 and AISI 316L has been studied because they have similar alloying elements (Fe, Ni, Cr, Nb, and Mo) and the same crystal structure. For this reason, good solubility is expected without the formation of complex intermetallic phases. Therefore, recent studies have been focused on developing bimetallic components with these materials using LP-DED [4,5,6,7,8,9,10].

For example, Xing et al. [6], evaluated the interface and mechanical properties of bimetallic IN 718–AISI 316L thin walls produced by LP-DED. The results showed a strong interdiffusion of alloying elements at the interface and some segregations near the Inconel 718 side. Ghanavati et al. [10] built IN 718–AISI 316L functionally graded materials. They showed that microsegregation of some alloying elements caused the formation of carbides and intermetallic compounds, affecting the interface properties. From the investigations described above, a series of issues are evident due to the differences in physical–chemical properties and the melting temperatures of each material. These differences distinctively influence the process parameters of melt pool, dimensional features, chemical composition, and properties. Furthermore, the fluid flow in the melt pool and the remelting of the previous layers can cause microsegregations, which give rise to the formation of secondary phases such as MC carbides and laves intermetallic compounds. For this reason, the selection of process parameters must be carefully controlled at all stages of fabrication.

Deep learning and machine learning approaches have been implemented to understand process–structure, process–properties, and structure–properties relationships due to the ability to find complex linear and nonlinear relationships between process parameters and study variables, providing accurate results from a previously developed dataset [11,12,13,14,15,16,17,18,19,20,21].

Previously, Choi [15] developed a data-driven approach, including implementing machine learning and deep learning algorithms to estimate the dimensional features of single scan tracks (SSTs), multitracks, and Cubes using AISI 316L, and implemented multiple linear regression, support vector machines (SVMs), gradient boosted regression, random forest, and artificial neural networks (ANNs) to estimate the height of the SSTs, multitracks, and weld angles. The ANN algorithm achieved high performance, achieving an accuracy of 85.37% for width, 81.59% for height, 96.63% for multitrack height, and 54.9% for weld angle. Lim et al. [14] developed a methodology to evaluate the relationship between process parameters and hardness, bead dimension, microstructure, and percentages of Ti, N, and O in a titanium alloy. Based on this information, a multi-classification model using the SVM-polynomial, SVM-radial basis function, and random forest algorithms was carried out. The random forest algorithm had the best accuracy of 96%. Furthermore, the SVM-radial basis function and SVM-polynomial had 85% and 82% accuracy, respectively. Kats et al. [12] developed a neural network linking the thermal conditions, including the thermal gradient and cooling rate, with the grain size and aspect ratio. The implementation of an ANN showed an MSE of 0.0149 for the loss and 0.0087 for the loss validation, describing the capacity to show complex relationships.

Although machine learning algorithms have achieved remarkable accuracy in the works described above, the application of machine learning in industrial applications can be challenging due to the number of variables and characteristics that directly and indirectly affect the structure and properties of the components [22,23]. Furthermore, previous works developed by Shin et al. [22] and Goodfellow et al. [24] highlighted the limitations of some ML algorithms in extending and re-training models when new data are added to the existing dataset.

Therefore, the aims of this research are twofold. The first objective is to understand the influence of the laser power and powder feed rate on the cross-section dimension, chemical composition, and microhardness of single scan tracks of IN 718 and AISI 316L on an AISI 316 substrate. The second goal is implementing a deep learning and machine learning algorithm to estimate and select the process parameters based on the best relationship between process parameters, dimensional features, chemical composition, and microhardness.

## 2. Materials and Methods

### 2.1. Powder Characterization

The metal powders used in this research are gas-atomized IN 718 and AISI 316L produced by Carpenter Additive (Philadelphia, PA, USA). The skeletal densities of the powders were evaluated using a Helium Pycnometer Ultrapyc 5000 (Anton Paar GmbH, Graz, Austria), and five measurements were carried out. Powder morphology was assessed qualitatively using a Phenom XL SEM scanning electron microscope (Thermo Fisher Scientific, Waltham, MA, USA) equipped with an energy-dispersive detector (EDS). Seven images were acquired at 300× to evaluate the particle size distribution. The chemical composition of both powders and the substrate are summarized in Table 1.

### 2.2. Experimental Setup

**Figure 1:**
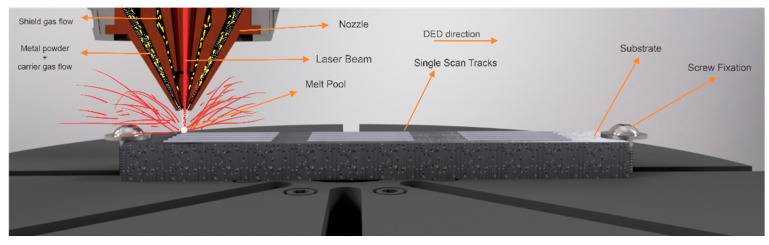
Experimental setup representation describing the process parameters, substrate, and SSTs.

The LP-DED system used in this research was a Modulo 250 manufactured by AddUp Solutions. It has an IPG photonic^®^ fiber laser (IPG Laser GmbH, Burbach, Germany) with a maximum laser power of 1 kW, a wavelength of 1070 nm, and a Gaussian spot size of ~60 μm. All experiments were carried out with 3.0 L/min carrier gas, 3.0 L/min central gas, and 6.0 L/min secondary gas. Both metal powders were deposited on an AISI 316 substrate. Figure 1 shows the experimental setup.

Table 2 describes the process parameters of the IN 718 and AISI 316L. These process parameters were selected from preliminary deposition tests, including evaluating possible burns, linearity, and oxidation. The SSTs were 40 mm in length.

In sequence, metallography preparation was carried out. The SSTs were cross-sectioned and prepared metallographically using SiC sandpaper for up to 2500 mesh and polished down to 0.02 μm. To reveal the cross-section and penetration, etching was employed using Kalling 2 solution for 15 s for the IN 718 and Marble etchant for 5 s for the AISI 316L.

### 2.3. Dataset Collection

**Figure 2:**
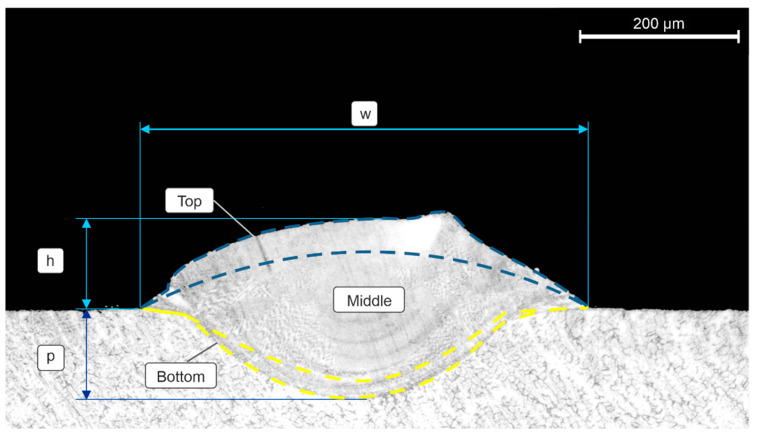
Representation of the cross-section describing the analyzed area.

Image acquisition and feature extraction were performed to evaluate the cross-section of the SSTs. Image acquisition was conducted using a Leica DMI5000 M optical microscope (Leica Microsystems GmbH, Wetzlar, Germany). The dimensional features of the SSTs were measured using the ImageJ Software (version: v1.51J8, National Institutes of Health, Bethesda, MD, USA), including the width (w), height (h), and penetration (p). Cross-sections were also analyzed using the EDS to evaluate the influence of the process parameters on the chemical composition. Three different regions (top, middle, and bottom) were delimited as described in Figure 2. The top and bottom areas were delimited by 70 μm from the upper/lower boundaries, respectively. In sequence, the five principal elements were analyzed.

Vickers microhardness tests were executed with a load of 300 g applied for 15 s. The microhardness was applied in the same areas described in Figure 2. Statistical bivariate correlations via Pearson correlations were used to understand the influences of the process parameters on the dimensional features and microhardness. This correlation coefficient was used in previous research to understand the linear bivariate correlation [25,26]. The Pearson correlation equation is given in Equation (1).

(1) $$r=\frac{\sum_{i=1}^{n}\left(X-\bar{X}\right)\left(Y-\bar{Y}\right)}{\sqrt{\sum_{i=1}^{n}{\left(X-\bar{X}\right)}^{2}}\;\sqrt{\sum_{i=1}^{n}{\left(Y-\bar{Y}\right)}^{2}}}$$

where *X* and *Y* are variable samples, $\bar{X}$ is the mean of the values in *X*, and $\bar{Y}$ is the mean in *Y*. To obtain an accurate result the metallography preparation, chemical composition, and hardness were calculated in three different cross-sections along the SSTs.

### 2.4. Deep Learning

The neural network model includes a feed-forward multi-layer perceptron for the regression. Equation (2) is the mathematical formulation for a single perceptron.

(2) $$y=\varphi \left(\sum_{i=1}^{n}w_{i}x_{i}+b\right)=\varphi (W^{T}x+b)$$

where *y*, *w*, *x*, *b*, and *φ* represent the outputs, the vector of weights, the vector of inputs, the bias, and the activation function. The model developed includes several dense layers interspersed with a nonlinear activation function (rectified linear unit (ReLU)) and has the ability to learn more complex patterns and avoid the vanishing problem observed in other functions such as sigmoid and tanh [27]. The model was compiled using the coefficient of determination (*R^2^*) and the mean absolute error (*MAE*) as a loss function shown in Equations (3) and (4).

(3) $$R^{2}=1-((\sum {\left(y_{i}-{\hat{y}}_{i}\right)}^{2})/(\sum ({y_{i}-\bar{y})}^{2}))\;$$

(4) $$MAE=\frac{1}{n}\;\sum_{i=1}^{n}\left|y_{i}-{\hat{y}}_{i}\right|$$

where *n*, *y_i_*, *ŷ_*i*_*, *ȳ* represent the sample size, the actual value, the predicted value, and the mean of the values in y, respectively. An adaptive moment estimation (Adam) was used to adjust the weight and bias, allowing the calculation of adaptive learning rates for each network parameter. The number of epochs used to train the model was 350, with 70% of the data for the training and 30% for the validation.

### 2.5. Classification

To select the optimal bead, we considered two evaluations acceptable: a dimensional feature height–width ratio (f) between 0.20 and 0.33 and a dilution (*d*) of up to 10%. The equation of the dilution is presented in Equation (5).

(5) d=p/h

Random forest was used to classify this task. This algorithm is used because of its performance in recent research compared with stochastic gradient descent and support vector machine algorithms [18]. The metrics used to evaluate the performance were *accuracy* (Equation (6)), *Precision* (Equation (7)), Recall (Equation (8)), and *F1-score* (Equation (9)).

(6) $$Accuracy=\frac{TP+TN}{TP+TN+FP+FN}$$

(7) $$Precision=\frac{TP}{TP+FP}$$

(8) $$F1\;score=2\left(\frac{Precision\times ecall}{Precision+Recall}\right)$$

(9) $$Recall=\frac{TP}{TP+FN}$$

where *TP*, *FP*, and *FN* are True Positive, False Positive, False Negative, respectively. These metrics are commonly used in classification models to evaluate the performance, as described in previous works [11].

## 3. Results and Discussion

### 3.1. Powder Analysis

**Figure 3:**
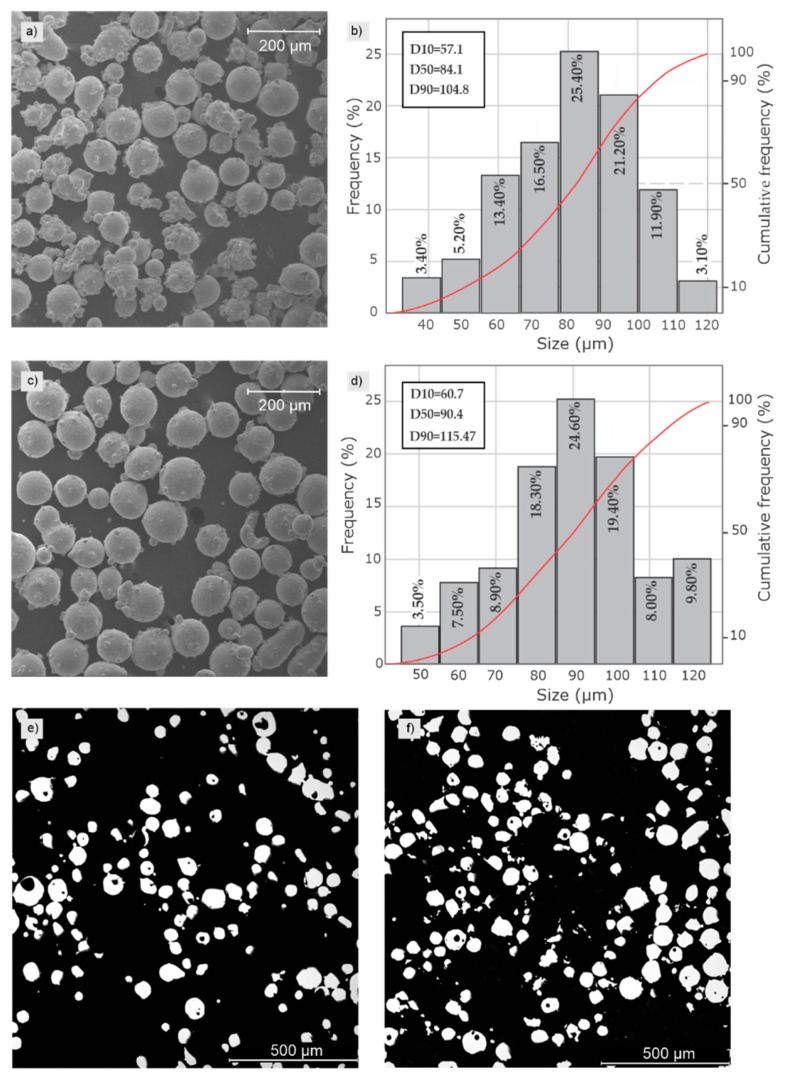
(**a**) SEM image and (**b**) particle size distribution of the IN 718 powder; (**c**) SEM image and (**d**) particle size distribution of the AISI 316L powder; and OM images of the powder cross-section for (**e**) the AISI 316L and (**f**) the IN 718 showing internal pores.

Powder analysis is a key factor in determining process parameters. Understanding particle morphology and particle size distribution allows for predicting the types of defects that may be observed during deposition, such as porosity related to the gas atomization process or lack of fusion related to powder morphology. Figure 3 presents micrographs of the powder morphology obtained via SEM and optical micrographs of the particles’ cross sections and particle size distributions measured using Fiji software (ImageJ2). Both powders contained mainly spherical particles, pores, some satellites, and a few elongated particles.

From the pycnometer results, the skeletal density obtained for the AISI 316L was 7.89 ± 0.03 g/cm^3^ and 8.18 ± 0.02 g/cm^3^ for the IN 718, indicating porosities of about 1.3% and 0.1%, respectively. Similar density values for powder obtained from the gas atomization process were observed in previous research [28,29].

### 3.2. Relation Process Parameters—SSTs Cross-Section Dimensions

**Figure 4:**
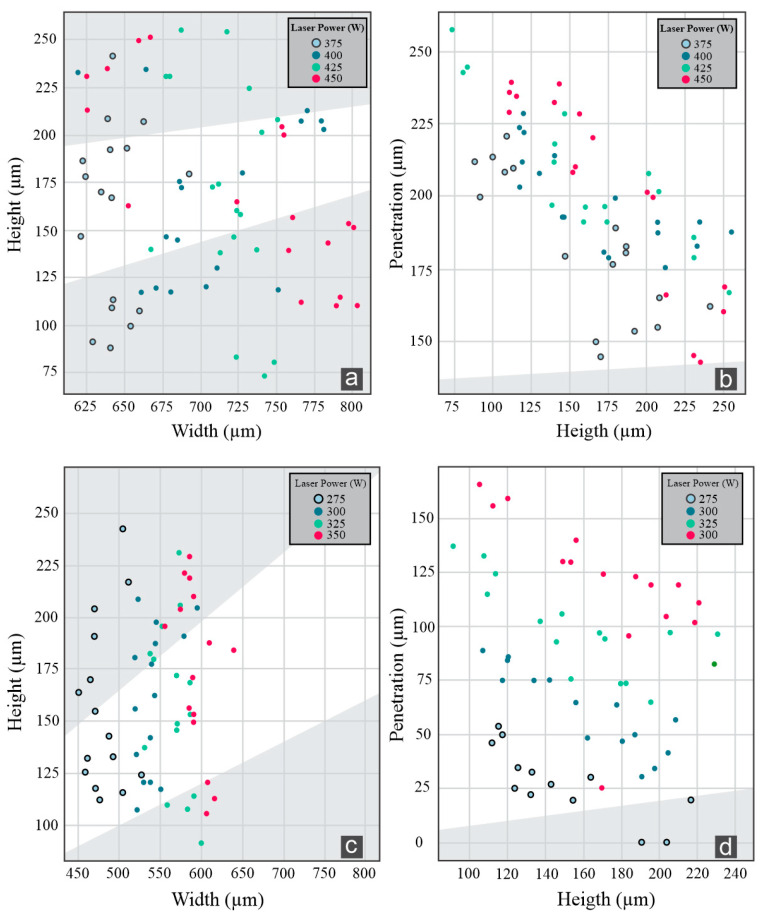
(**a**) Height–width ratio and (**b**) dilution of the IN 718 SSTs on the AISI 316 substrate, and (**c**) height–width ratio and (**d**) dilution of the AISI 316L SSTs on AISI 316 substrate.

The fabrication of SSTs is essential to achieve a range of optimal process parameters when a new metal powder is used [30]. When the SSTs are deposited, visual inspection is the first step to identifying possible burns or non-linearity. This is crucial for designating the boundary limits of the process parameters for each material used. All beads showed linearity from visual inspection, and no burning was observed. From the criteria described in Section 2.4, the IN 718 showed good quality, with powder feed rates of 3.0 g/min and 3.5 g/min with laser powers of 400 W, 425 W, and 450 W, and 4.0 g/min with laser powers of 425 W and 450 W. For the AISI 316L, powder feed rates of 3.0 g/min, 3.5 g/min, and 4.0 g/min with laser powers of 275 W, 300 W, 325 W, and 350 W, showed good quality, and for 4.5 g/min the laser powers were 275 W, 325 W, and 350 W. The influence of the process parameters in the dimensional features described different behavior for each material, as described in Figure 4.

**Figure 5:**
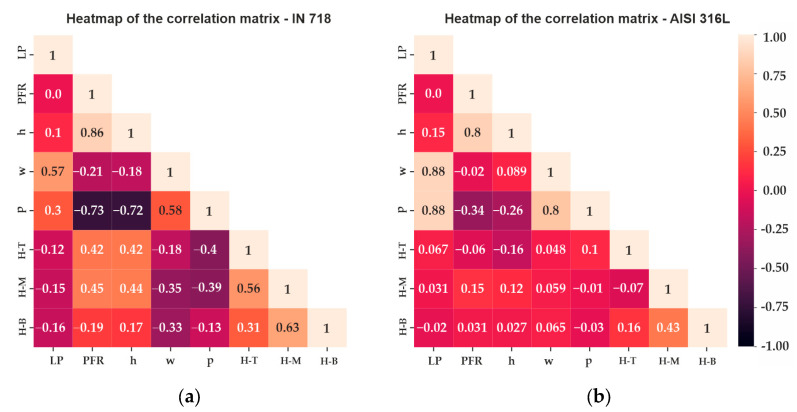
Heatmap of the Pearson correlation for the (**a**) IN 718 SSTs on the AISI 316 substrate and (**b**) for the AISI 316L SSTs on the AISI 316 substrate. (H-B) Hardness Bottom, (H-M) Hardness Middle, (H-T) Hardness Top.

Evaluating the correlation between the process parameters with dimensional features for the IN 718, the laser power (LP) showed a moderate correlation with the width (0.57) and the penetration (0.30) and a weak and positive correlation with the height (0.10). The powder feed rate (PFR) showed a high correlation with the height (0.86), a weak and negative correlation with the width (−0.21), and a high and negative correlation with the penetration (−0.73). The Pearson correlation values are presented in a heat map in Figure 5.

Regarding the AISI 316L SSTs, a different pattern was observed. The laser power showed a high and positive correlation with the width (0.88) and the penetration (0.88) and a weak and low correlation with the height (0.15). In contrast, the powder feed rate showed a high and positive correlation with the height (0.80), a weak and negative correlation with the width (−0.02), and a moderate and negative correlation with the penetration (−0.34). The correlation values of the laser power with the penetration and width showed the same behavior in recent studies with different AISI 316L powder [31].

### 3.3. Relation Process Parameters—Chemical Composition

Evaluating the chemical composition in bimetallic tracks is critical to understanding how laser power and adding metal powder can reduce the diffusion of the alloying elements in the IN 718 and AISI 316L SSTs. From this approach, for the IN 718, it was possible to observe three cases:

- Low powder feed rate (2.0–2.5 g/min)
- Medium powder feed rate (3.0–3.5 g/min)
- High powder feed rate (4.0–4.5 g/min)

**Figure 6:**
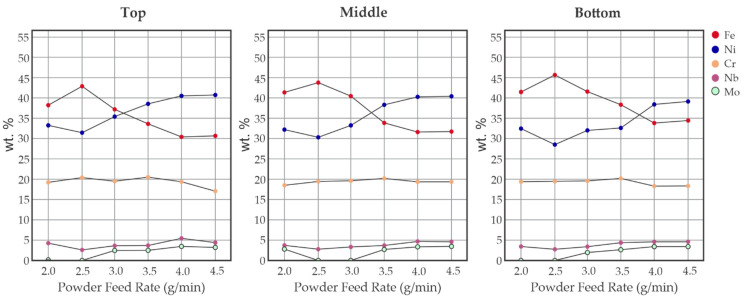
The evolution of chemical composition in IN 718 SSTs on an AISI 316 plate. Laser power = 450 W.

For the low powder feed rate, predominantly high Fe levels were observed in the three regions analyzed, as reported in Figure 6 for an LP = 450 W. The values of Fe for the bottom, middle, and top for the 2.0 g/min rate were 42%, 41%, and 39%, respectively. These values increased when the powder feed rate increased to 2.5 g/min to 46%, 44%, and 43%, respectively. In the medium powder feed rate, the Fe percentual decreased progressively. As the powder feed rate increased to a medium powder feed rate the values of Fe decreased, obtaining a change of the main element alloy at about 3.5 g/min for the top and middle, and between 3.5 g/min and 4 g/min for the bottom region. In a high powder feed rate, specifically at 4.5 g/min, the values of Ni for the bottom, middle, and top were 39%, 40%, and 41%, respectively. This trend was expected due to the amount of Ni and alloying elements that are added to the melt pool when high PFRs are used. This trend and the Pearson correlations presented in Figure 5 confirm this finding, with a moderate correlation between the powder feed rate and the Vickers microhardness for the middle and top regions.

It is interesting to underline that the transition where the Ni content becomes higher than the Fe content begins at the top of the bead when low PFRs are used and shifts to the bottom of the bead as the PFR increases. This suggests that the fluid flow in the melt pool is insufficient to fully homogenize the composition, leaving the deposited alloying elements, i.e., Ni, Mo, and Nb, mainly located in the top part of the deposited track.

The Cr values observed varied in the 18% to 20% range; the Nb content increased as the powder feed rate increased from 2.5% to 5%. Mo followed the same behavior on a lower scale between 0% and 3.5%. As expected, the composition of AISI 316L SSTs is not affected by the powder feed rate and it is always in the ranges reported in Table 1. This is due to the similar chemical composition of the material and the substrate.

### 3.4. Relation Process—Microhardness

**Figure 7:**
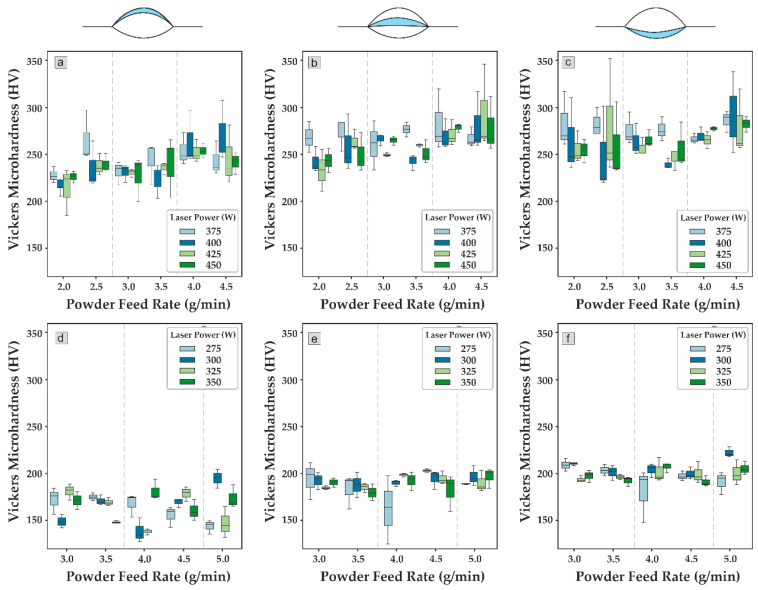
Boxplot of Vickers hardness at the (**a**) top, (**b**) middle, (**c**) and bottom of the IN 718 and (**d**) top, (**e**) middle, and (**f**) bottom of the AISI 316L.

Figure 7 shows a boxplot of hardness vs. powder feed rate and laser power. The values of microhardness indicate the influence of diffusion on mechanical properties. For IN 718, a low powder feed rate showed high variability in the bottom area, with values between 220 HV and 352 HV. This variability decreased in the middle and top regions, with values between 211 HV and 293 HV for the middle area and between 185 HV and 297 HV for the top region. The hardness levels increased as the powder feed rate increased, while variability reduced. For medium powder feed rates, the values observed at the bottom were 233 HV to 295 HV, in the middle 233 HV to 286 HV, and in the top 200 HV to 265 HV. In the case of the AISI 316L, less variability was observed when compared with the IN 718. The bottom region showed values between 147 HV and 228 HV, and the middle region values between 124 HV and 211 HV. In the case of the top, the values obtained were 126 HV and 203 HV.

On the other hand, for 316L, the hardness remained with low variability across the entire parameter range. As observed from the Pearson correlation, the process parameters did not affect the microhardness. It is well known that the AM process parameters affect the cooling rate and, consequently, the microstructure and properties such as microhardness of the build part. However, in the parameter range used in this study, selected as it allows for good quality tracks, no influence of laser power and PFR on hardness linearly was detected.

### 3.5. Deep Learning Results for the Regression Task

**Figure 8:**
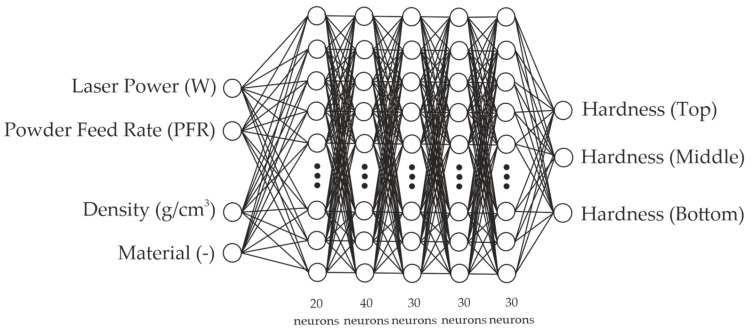
Representation of the network architecture of the three sets.

The architecture developed in this work was composed of four input parameters, including laser power (W), powder feed rate (g/min), skeletal density (g/cm^3^), and material; five hidden layers with 20, 40, 30, 30, and 30 neurons, respectively. The outputs were divided into three sets: the first included dimensional features (h, w, and p), the second included the chemical composition at the top, middle, and bottom (15 outputs), and the third included the microhardness at the top, middle, and bottom (3 outputs). A representation of this architecture for set 3 is described in Figure 8.

Understanding the value of MAE and R^2^ obtained from the model allows us to evaluate how the network can improve the estimations from the dataset provided. For this reason, assessing MAE and R^2^ for loss and loss validation is crucial for avoiding underfitting and overfitting [32]. The criteria used to evaluate the number of hidden layers of the model was the convergence of the loss and validation loss, the stability of both curves with loss and validation loss values close to each other, and validation loss values slightly higher than the loss values. Additionally, a determination coefficient close to 1 was considered an important factor in evaluating the model’s performance.

Table 3 presents the MAE values for the loss and validation loss. We can observe high MAE values for the loss and validation loss in the first 100 epochs. The model described a convergence before the 150 epochs and kept the proportion of loss down for the validation loss values, indicating a good learning rate.

Comparing the loss and validation loss in all architecture described a good fit. Overfitting was not observed.

### 3.6. Machine Learning Results for the Classification Task

The good beads were selected based on the criteria described in Section 2.5. The model described an equilibrated performance between good quality predictions and bad quality predictions. The model’s overall accuracy was 80%, meaning that 80% of the predictions were correct. The precision allows us to understand the proportion of predicted positive cases that are correctly real positives; recall is the proportion of real positive cases that are correctly predicted positive [33]. Based on these two metrics, we can observe a precision of 85% for good quality predictions, 75% for bad quality predictions, and a recall of 78% and 83% for good and bad quality predictions, respectively. These results suggest that the model slightly favors identifying bad quality predictions correctly over good quality, despite achieving higher precision for good quality predictions. The details of the classification are reported in Table 4.

The macro average explains how the classifier improves in general terms without considering the class imbalance. Instead, the weighted average considers the imbalance of the classes, giving more weight to the majority class [34]. In both cases, we can observe that the macro average and weighted average values for precision, recall, and F1-score are quite close, which indicates that the model is performing consistently on both metrics without considering the imbalance and considering it.

## 4. Conclusions

In this study, the influence of the laser power and powder feed rate in the dimensional features, chemical composition and microhardness of the IN 718 and AISI 316L SSTs on an AISI 316 substrate have been investigated, and a deep learning and machine learning approach has been implemented to estimate and classify the quality of the beads. From the results obtained, we can highlight the following conclusions:

- In both materials, the laser power directly influences the width and penetration of the beads while the powder feed rate mainly affects the height of the beads.
- The PFR directly affects the chemical composition of the IN 718 beads, while, due to the chemical composition close to the substrate one, it does not affect the AISI 316L ones.
- The deep learning architecture demonstrated a good fit, as highlighted by the behavior of the loss and loss function over the 350 epochs. The convergence of the loss and validation loss function was from epoch 150.
- The classification approach was able to classify the quality of the beads with an accuracy of 80%. The results of the macro average and weighted average described values quite closely considering the imbalance and without considering it.

## Figures and Tables

**Table 1:** Chemical composition of AISI 316L, AISI 316, and IN 718.

Element (wt.%)	Fe	Cr	Ni	Mn	Mo	Si	P	S	Nb + Ta	Al	Ti	C
AISI 316L	Bal.	16–18	10–14	<2.0	2–3	<0.75	≤0.04	≤0.03	-	-	-	≤0.03
AISI 316	Bal.	16–18	10–14	<2.0	2–3	<0.75	0.04	≤0.03	-	-	-	≤0.08
IN 718	Bal.	17–21	50–55	<0.35	2.8–3.3	<0.35	<0.01	<0.01	4.75–5.56	0.2–0.8	0.65–1.15	<0.08

**Table 2:** Process Parameters.

Material	Laser Power (W)	Powder Feed Rate (g/min)	Scan Speed (mm/min)	# of SSTs
IN 718	375–400–425–450	2.0–2.5–3.0–3.5–4–4.5	2000	24
AISI 316L	275–300–325–350	3.0–3.5–4.0–4.5–5.0		20

**Table 3:** The MAE and R2 values for the loss and loss validation for the three sets after 350 epochs.

Set	Dimensional Features		Chemical Composition		Microhardness	
Epoch	Loss	Loss Validation	Loss	Loss Validation	Loss	Loss Validation
25	282.46	282.10	16.83	16.56	217.06	210.02
50	200.47	194.83	7.46	7.63	78.45	75.05
100	38.79	41.65	3.22	3.25	18.91	19.50
150	30.57	33.99	2.38	2.43	14.20	14.80
200	29.31	32.12	2.33	2.35	13.01	14.31
250	29.61	31.93	2.36	2.33	12.99	14.21
300	30.22	32.04	2.24	2.32	12.89	14.13
350	29.57	31.64	2.20	2.33	13.07	14.36
R^2^	0.97	0.96	0.94	0.94	0.82	0.79

**Table 4:** Classification report on the quality of the bead.

	Precision	Recall	F1-Score	Support
Bad Quality	0.75	0.83	0.79	29
Good Quality	0.85	0.78	0.82	37
Accuracy			0.80	66
Macro avg.	0.80	0.81	0.80	66
Weighted avg.	0.81	0.80	0.80	66

## Data Availability

The original contributions presented in the study are included in the article. Further inquiries can be directed to the corresponding author.
